# La Charite

**Published:** 1829-04-01

**Authors:** 


### LA CHARITE.

CASE OF EPILEPSY OF 14 YEARS' STANDING—HÆMORRHAGES FROM THE MUCOUS MEMBRANES—SEIZURE OF THE REIGNING EPIDEMIC—CURE OF THE EPILEPSY.

A female, aged 55 years, had daily attacks of epilepsy since the year 1814. These attacks were ascertained during the first days of her residence in the hospital. She had two seizures every 24 hours. For several years past, she was subject to discharges of blood from the bowels, and to considerable uterine losses. Yet she was embonpoint—the digestion unaffected—she had no appreciable disease of the uterus. She had been bled copiously, without any relief of the epileptic paroxysms. Ten days after coming into the hospital, she complained of violent pains in the soles of the feet—the whole of her skin became the seat of severe prickings—and, in short, she exhibited the usual symptoms of the Parisian epidemic already described in the last number of this Journal, (p. 156.) The epilepsy disappeared! The epidemic affection lasted twelve days, during which she was free from epilepsy. A new series of phenomena now commenced—a succession of hæmorrhages, alternately from the mucous membrane of the nose, fauces, stomach, bronchia, vagina, and rectum. Nevertheless, the various organs from whence the blood issued, shewed no other symptom of derangement except the hæmorrhage. The epidemic disease ceased—the hæmorrhages ultimately disappeared—and the epilepsy did not return. The patient was discharged the hospital apparently cured.

The patient will be fortunate indeed if she escape a re-visitation of the epilepsy. But we fear this will not be the case.

II. SUCCESSION OF MORBID PHENOMENA IN AN INDIVIDUAL.

A young man had had numerous boils on different parts of the body, the vestiges of which were very evident. When received into LA CHARITÉ, he had well-marked urticaria. This disappeared, and was succeeded by acute rheumatism, affecting three joints of the upper extremities. Tartar-emetic, in large doses, was given; and he was bled. In five or six days the rheumatism ceased—and, on the following day, erysipelas appeared on the face—ran through its phases without any bad symptoms—and required no active treatment. When the patient was convalescent, he, one day, complained of some pain in the left scapula, where there was some swelling, but no redness. Here an enormous abscess quickly formed, with so little pain that he could lie on his back till the abscess was opened. Some people will call this abscess critical—and so may be denominated the perspiration in the following case of dropsy.

III. ASCITES CURED—APPARENTLY BY PERSPIRATION.

A young female, after having evinced all the symptoms of peritonitis, became affected with ascites, which, in a short time, increased to a very considerable size. The lower extremities swelled—the face became œdematous—the urine scanty. Digitalis, squills, and various diuretics, were employed, without success—and the operation of paracentesis was deemed inevitable. At this time the patient mentioned that she had a profuse perspiration, though previously her skin was very dry. The legs now became smaller. The diuretic medicines were suspended—the urine became more copious—the abdomen diminished in size—the night sweats continued—the dropsy entirely disappeared—and the patient left the hospital cured—thanks to Nature rather than the doctors! In the following case the scene is reversed—and ART is said to have accomplished what NATURE failed to do.

IV. ACUTE RHEUMATISM—INUTILITY OF VENESECTION—EMPLOYMENT OF LARGE DOSES OF TARTRITE OF ANTIMONY—OPIATES.

A female was admitted, under severe pain in the soles of the feet, which she compared to that produced by the implantation of pins or needles. There was no fever—no disorder of the digestive functions. This woman had had the reigning epidemic of Paris. In a day or two, the ankle became inflamed and swelled—fever was lighted up—and acute rheumatism was established in the place of the epidemic. *Venesection—twenty leeches.* Next morning the inflammation had departed from the leeched ankle, and fixed itself on one of the wrists. *Another venesection—another leeching.* The inflammation now quickly migrated to the other wrist, leaving the one first affected. Another bleeding was practised;—and, in 24 hours after this last depletion, several joints became swelled and inflamed, the fever getting very high. Eight grains of tartar-emetic were now dissolved in four wine-glassfuls of a vegetable infusion, and half a glass was administered every two hours. This produced abundant vomiting and purging. In the evening, every trace of rheumatism was gone; but the relief was only temporary. The rheumatism returned in 30 hours, and several joints were simultaneously affected. The fever was now very intense. Thus the bleedings, the leechings, the Rasorian employment of antimony, had all failed. In this case, M. Andral had recourse to opium, both internally and externally. The effects were like magic. Next day there was not a trace of pain or fever. The opium was diminished—convalescence was rapid—and the patient was soon discharged cured.—*Journal Hebdomadaire.*

The last case exemplifies a position we have not neglected to lay down for many years past—namely, that acute rheumatism is not to be cured by bleeding. We verily believe that, upon the whole, the disease is more frequently exasperated than relieved by it. When the pain and fever run high—the constitution is sound—and the patient young or vigorous, a moderate bleeding may do some good—rather by promoting the operation of other medicines, than by any intrinsic efficacy of venesection itself. In cases, too, where metastasis to an internal organ takes place, we must bleed. But, under opposite circumstances, beware of the LANCET—we do not mean that droll *blade* sold at eight-pence in the Strand—but those "minute instruments of mighty mischief," which Weiss, Simpson, Stoddart, and some others in the same street, supply to our brethren, at the extravagant price of 2s. 6d. each.

V. CASE OF STRANGE AND ANOMALOUS NERVOUS SYMPTOMS IN A FEMALE, IMITATING ORGANIC DISEASES AND DISAPPEARING ON THE ERUPTION OF THE CATAMENIA.

Elizabeth Babillon, aged 37, a widow, entered LA CHARITÉ on the 14th of May, 1828. This woman was the mother of five children—had been healthy—regular—and strong, till some mental afflictions and misfortunes disturbed her health, and even her reason. For this last derangement she had been some time in the SALPETRIERE. When received into LA CHARITÉ, she complained of violent palpitations, tightness of the chest, pain in the left side—symptoms which she traced to a fall which she had met with 18 months previously, at a time when she was in the habit of being bled every two months. On examination, the pulse was found to be irregular and intermittent—the beating of the heart was heard over a considerable space—no œdema. The patient was considered as highly nervous, by M. Fouquier—was bled, leeched, and placed on very rigid regimen. The cardiac symptoms very speedily abated; but now the patient complained of violent pain in the head, chiefly on the right side, which was relieved by the patient applying cold water to the head in the night. Whenever the head-ache was temporarily relieved, she felt distressing numbness and stiffness in her limbs. In the course of a short time, there was complete paralysis of the right side, both of sensation and motion. Acupuncture on that side excited no sensation. *Sinapisms—blisters.* In the beginning of September, the right lower extremity, hitherto paralytic, became completely rigid, and remained in a state of permanent extension. When flexion was forced, great pain was produced. Both thumbs, at this time, became rigidly extended, and there was very little sensibility in either of the arms. M. Andral now succeeded M. Fouquier, and applied moxas to the loins, which almost immediately restored sensibility to the lower extremities. But now a most intense pain was felt in the region of the heart, radiating thence along the left arm. There was no fever — no convulsions. Next day (11th September) a sense of constriction was felt in the throat—deglutition became difficult—and the voice was nearly extinct. Passing over some other metamorphoses of the sly Proteus Hysteria, we find the menses appearing on the 15th September, when the whole of the teasing phenomena above-mentioned vanished like a dream!—HEBDOMADAIRE.

There was not the slightest reason to suppose that this female had any intention to impose on her medical attendants. She could have no possible inducement so to do; and the moment she was relieved of her sufferings, she acknowledged the relief. We do not believe there was any deception. If hysteria can imitate WHITE SWELLING of the KNEE, it may imitate paralysis, organic disease of the heart, and any other ill to which flesh is heir. We have recently observed it to put on the appearance of *diabetes*—not diabetes insipidus, for that is a common form of hysteria; but diabetes *mellitus!* We perfectly agree, therefore, with M. Andral in the following sentiment. "Instead of amalgamating the phenomena exhibited by this female with the paralysis resulting from effusion on, or disorganization of the brain, we should assimilate them with those temporary hemiplegiæ, those partial paralyses which we meet with in persons of an hysterical character." In short, if there is any one class of diseases which requires a new and a more extended investigation, it is the class of hysterical affections under anomalous forms!

VI. SPINAL DISEASE.*

That many patients have been confined to hard boards and hair mattresses by the Spine-doctors of town and country, whose spines and spinal marrows were perfectly sound, there can be no doubt; and this spino-mania has now produced a natural re-action in the public mind, which is likely to sally too far towards the other extreme—that of over-looking real spinal diseases, and considering them as nervous, hysterical, or sympathetic affections of no serious importance. The following case is deserving of attentive perusal.

*Case.* N———, porter of the Polytechnic School, aged 50 years, of melancholic temperament, had been ailing some months prior to his reception into LA CHARITÉ, which was in the beginning of June. He walked to the hospital without assistance, and, on examination, no particular disease could be detected, with the exception of pain between the shoulders. It was even proposed to discharge him from the hospital, when he suddenly became affected with severe pain in the lower extremities, and along the spine. This was soon followed by complete paralysis of the former, and total inability to expel either fæces or urine. The breathing became laborious, and the patient died in the course of eight days after his reception into the hospital.

* *Journal Hebdomadaire.*

*Dissection.* There was no external appearance of disease, or deviation from the natural form of the spinal column; yet, on removing the muscles, two or three vertebræ were found carious, in their spinous processes, with suppuration around them. These were the upper dorsal vertebræ. Corresponding with these, the spinal marrow was found softened, even to diffluence; and its substance mixed or incorporated with purulent matter. For about six inches above and below this softened portion, the medulla spinalis was red, and evidently inflamed. The meninges were also unequivocally inflamed, and, in some places, lined with a layer of purulent matter. The bodies of the vertebræ were not carious. The meninges of the brain were inflamed aud thickened. There were some depôts of purulent matter in the substance of the lungs—and some purulent effusion into the cavity of the pleura. The left ventricle of the heart was in a state of hypertrophy. The right kidney was twice its natural size, and "transformed into a large bag filled with purulent matter, and lined with a smooth membrane, similar to that which exists in some cases of pulmonary excavations." The other kidney was also in a state of disease, and contained purulent matter.

Such extensive disorganizations in a patient whom the medical officers were inclined, a few days previously to death, to discharge from the hospital as an impostor, led to some inquiries respecting the history of the complaint, and M. Gualtier de Claubry, who had attended the man in the Polytechnic School, furnished the following particulars. The patient had long complained of pains in his back, which he considered rheumatic. During the last six months these pains had increased, to which were added numbness in the lower extremities, and obstinate constipation. Eight days before he was conducted to the hospital, the patient could hardly walk, and he often fell down in the attempt. The legs and thighs were sensibly emaciated, and he complained of a dull pain in the dorsal region of the spine, which was increased by pressure there. From that point there was an irradiation of pains in various directions. He had great difficulty in voiding his urine and fæces. M. de Claubry, on examination, discovered a tumour in the right side of the abdomen, and, as he thought he could distinguish a pulsation in it, he concluded that it was aneurismal. This was, of course, the enlarged kidney.

The foregoing case ought to put us on our guard against hasty conclusions—or rather against laziness in the examination of diseases. Of the innumerable *false facts* which have been put upon the records of medicine, and which have greatly tended to retard its progress as a science, we believe that a great proportion have been occasioned by want of accuracy in observation or investigation, rather than by *mala fides* in recording the particulars! Want of attention is certainly less *culpable* than want of veracity—but it is hardly less *excusable*, in a science where health and even life is concerned. Many serious errors might be avoided—many disagreeable exposures shunned, by a few minutes' additional attention to the investigation of existing symptoms, and inquiry into the previous history of the case. The increasing competition in every department of the profession, and the daily augmenting ratio of scientific acquirements in its members, will force this *attention* to individual cases, if men wish or hope to keep their station in medical society.

### VII. WOUND OF THE HEART—PERICARDITIS—LIFE SUSTAINED FOR NINE DAYS.

Victor Janson, aged 16 years, while wrenching a knife, in play, from a comrade, struck its point against his own chest, which it penetrated rather deeply in the region of the heart. He felt no pain at the moment, and did not even think that the point of the knife had gone farther than through the clothes. In about ten minutes, however, he perceived blood issuing from his side, which was soon followed by sickness and vomiting. His comrade bound something over the wound; but the blood continued to flow, and the patient fainted. A medical man was called, who applied some compresses, and Janson was immediately conveyed to LA CHARITÉ. When received, (8th September,) his face was pale, extremities cold, respiration short, pulse quick, small, and weak, much blood on the shirt and clothes. The compresses being removed, a wound was observed between the 4th and 5th ribs, two or three fingers' breadth from the edge of the sternum, and about six or seven lines in extent. The blood was not flowing at this moment, but on the patient's making a slight movement, it spouted out, and soon again ceased. It was observed that the wound of the skin did not correspond with that in the intercostal muscles—a circumstance that prevented the blood from issuing uniformly. A probe was introduced, and it was evident that the knife had penetrated the thorax, and that, in all probability, the heart itself was wounded. Dressings were applied, and the patient put to bed on his back. With great difficulty eight ounces of blood were abstracted from the arm. The left side emitted a dull sound, and the respiration was very feeble. The right side was quite the reverse. These examinations indicated effusion of blood into the left cavity of the pleura. 9th. The patient got some sleep in the night—felt a good deal of oppression—some hæmorrhage from the wound—pulse risen—re-action coming on. M. Boyer prescribed three bleedings, with a few hours' interval. The first venesection (12 oz.) produced momentary relief—the second and third (same quantities) diminished the oppression. *Gum-water for drink—no food.* 10th. No sleep last night—breathing quicker—pulse stronger. Venesection, which was repeated in the evening, the symptoms being very distressing. 11th. Great agitation during the night, with inclination to vomit—pain in his head—great debility. The dressings were removed by M. Boyer, and the blood spouted forth. The left side sounded very badly—and appeared rather bulged out. The dressings were replaced, and the patient seemed a little relieved by the discharge of blood. In the evening, the oppression was great, the breathing quick. Twenty leeches to the anus. 12th. The head-ache and tenderness of abdomen almost gone; but the anxiety and oppression had increased—pulse 144 in the minute—fever great. There was a grating noise heard, and even felt with the hand, in the region of the heart. 13th. The symptoms nearly the same as yesterday—a fresh hæmorrhage, after some attempts to cough. The feebleness of the pulse contra-indicated any farther depletion, and revulsives only were applied to the extremities. He denied having any pain in the region of the heart. 14th. The oppression being very great to-day, M. Boyer introduced a probe into the chest, and blood spouted out to the distance of several feet. M. Boyer withdrew the probe, and endeavoured to introduce his finger, which brought on a convulsive action of the respiratory muscles, and a still more forcible eruption of black blood. Some air got into the chest both times. The oppression was diminished for a very short time, but was soon again urgent, with attempts to vomit, and renewed hæmorrhage. In the night, the poor boy's sufferings were dreadful. He lingered out till the 17th September, when he expired, shortly after a considerable hæmorrhage from the wound.

*Dissection.* M. Boyer made an opening into the inferior parts of the left side of the chest, when about a pint and a half of blood escaped. The thorax was then opened by raising the sternum, when the pericardium was observed to be punctured, thickened, and inflamed. It adhered to the heart near the wound, and pus was seen in its interior, of a thick and green colour. A corresponding wound, though smaller in size, was discovered in the parietes of the left ventricle of the heart, through which a probe easily passed into the cavity, permitting a little clot of blood to escape. The internal orifice of the wound was observed to be very narrow, the point of the knife having merely penetrated it. The internal surface of the pericardium, and the external surface of the heart were covered with false membranes. The tissue of the heart itself had begun to inflame, being thickened and indurated. There was no extravasation of blood within the cavity of the pericardium—probably on account of the prompt adhesion, near the wound, of the pericardium to the heart. The lungs on both sides were sound. Nothing is said of the state of the pleura in the left side of the chest. The blood had flowed out of that side when the incision was first made between the ribs.—Journ. Hebdomadaire, No. 1.

### XXXII.

### LA CHARITE.

#### I. PHLEGMATIA DOLENS IN A MALE.

As the pathology of this disease is still *sub judice*, we ought to examine with candour and discrimination each fact that comes before us well authenticated.

Henry Fouques, aged 15 years, was placed in the ward St. Louis of the above hospital, on the 7th July, 1828. He dated his malady only from the preceding day, and could assign no cause for the complaint. He had now acute pain in the right groin, augmented by pressure; and in that part was felt a small tumour. The abdomen was slightly painful, and there was some diarrhœa—tongue white in the middle, and red at the point—cephalalgia—pulse hard and frequent—heat of skin—thirst. *Diluents—lavements.* 8th. The general symptoms remain the same; but other phenomena were developed. At the lower and inner side of the right leg, there was an erysipelatous redness, to the extent of a hand's breadth, from whence stripes of redness ascended up to the groin. These were extremely painful on the slightest pressure—the inguinal glands were now unequivocally enlarged—the thigh and leg were swelled, tense, and did not leave any pit on pressure. The patient dared not move the limb in the slightest degree without the most acute pain. *Venesection to* 12 *ounces—fomentations—lavements, diluents.* 9th. The red stripes are of a more vivid colour, and more clearly defined. They are very painful. They did not follow the course of the internal saphena vein, which was neither hard nor tender to the touch. The constitutional symptoms remained the same. The blood drawn was inflamed. *The venesection repeated.* 10th. The blood last drawn was not inflamed. The local symptoms are diminished. The redness of the stripes, and the swelling and tension of the whole limb, are less—the inguinal glands are smaller. *Bled to six ounces.* 11th. The blood again inflamed. Large phlyctenæ have formed on the lower part of the leg. 12th. "The extreme pain felt in the course of the lymphatic vessels that were inflamed is nearly gone." From this time the general and local symptoms declined, and the patient was discharged cured, with the exception of some œdema of the limb, which still remained when he left the hospital.

M. Littre, who reports this case, under M. Lerminier, considers it one of phlegmatia dolens, and that the inflammation was seated in the lymphatic glands and vessels. There certainly is a great similarity in many of the symptoms; but the red lines running in the direction of the absorbents are not usually seen in the puerperal disease. The tension and elasticity of the swelling—the great pain in attempting to move the limb—and the want of pitting after pressure, are all features of the phlegmatia dolens of authors. That the inflammation, in this case, was not seated in the veins, is as certain as any thing can be, where dissection did not take place.—*Journal Hebdomadaire.*

II. HÆMORRHOIDS—MODE OF OPERATING AT THE ABOVE HOSPITAL.

In this country, surgeons, or at least the more experienced and eminent amongst them, have abandoned the operation of excision, in consequence of the dangerous bleedings to which it has now and then given rise. Sir Astley Cooper has publicly protested against the operation, and Mr. Brodie has for many years given it up. The usual mode now employed, is to pass a double ligature through the base of the pile, each half of which is separately tied, as tight as the surgeon can draw the silk. The portion of the pile above the ligature is then removed by the knife or scissors, without much pain or any possibility of bleeding. Mr. Brodie, whose experience in these cases has been very extensive, never saw the slightest ill consequence follow the operation thus performed, nor are we aware of any having been recorded.

At La Charité, a woman, who had suffered from internal piles for many years, was operated on by M. Roux in the following manner. Three clusters, varying in size, from the volume of a nut to that of a cherry, were transfixed at their base by a curved needle carrying a thread, which was left in, in order to prevent the rectum being wounded when the tumours were cut. The probe-pointed bistoury was then employed, to dissect out the pedicle of the tumours—several arteries which bled were tied—and the protruding rectum was returned. Compresses smeared with ointment, and tied round the middle with thread, were stuffed into the rectum, and the anus filled with dossils of lint. The patient went on well, no hæmorrhage took place, and, after three days, the dressings were removed.

The reporter remarks that a better mode of stuffing the rectum is adopted at the Hôtel Dieu. It consists in a linen bag, through which there passes an elastic gum catheter, whilst the space between the external side of the instrument and the wall of the bag is filled with lint. The catheter allows the stools (liquid ones) to pass, whilst the stuffing of the bag makes compression against the sides of the gut. The contrivance is ingenious, and, on other occasions, the hint might be turned to account, but, indeed, it is totally unnecessary in the English manner of operating for piles. If we may judge by the details of the above operation, our neighbours are far behind us in their surgical treatment of hæmorrhoids.

### CLINICAL REVIEW.

### LIV.

### LA CHARITE.

### On Hypertrophy of the Brain. By M. Laennec, M.D.

This is a disease which is little known to pathologists and still less to nosologists. The late celebrated Laennec (cousin to the author of this paper) was the first, we believe, to draw the attention of physicians to this subject; but, since his observations were published, several others have been made known by Dr. Dance, Professor Scoutteten, and the author under review. Morgagni, indeed, had remarked that, in some bodies which he had opened, the brain seemed to be too large for the skull, the latter appearing to have compressed the former, (Epis. 4.) M. Jadelot had observed the same thing in many children who had died with symptoms of hydrocephalus, but who, on dissection, presented no other pathological condition than this disproportion between the brain and the skull. The author of the great work on auscultation had occasion to see several cases where the disease was supposed to be hydrocephalus, and where, when the head was examined, a very small quantity of water was found in the ventricles, while the convolutions of the brain were flattened very much, and, consequently, where the brain must have undergone considerable pressure before death. These observations were published by Laennec so long ago as 1806; and in the year 1823, in a discourse before the College of France, M. Laennec made use of the following expressions:—

"This alteration (says he) is common in children, but more rare in adults. It is characterised by a very great firmness of the cerebral substance, and by a considerable flattening of the convolutions, although the ventricles contain but very little serum. It is sometimes developed very slowly; but more commonly it presents itself as an acute affection, the symptoms of which are very analogous to those of hydrocephalus. It does not appear to be the result of inflammation, nor of excessive action in the brain; for it is not more common to men of great study than to others. Inflammation, indeed, does not seem to be the cause of hypertrophia in any organ of the body, if we except the tonsils. In almost all the other organs, prolonged or frequently repeated inflammation ends by producing *atrophy* of the part."

Although the latter part of the above passage may not have gained general assent, yet even this short notice excited attention from others. In the *Archives*, for January, 1825, M. Scoutteten has related an interesting case bearing on the subject in question. A child, aged five years and a half, whose head was very large, but whose intellects were very feeble, died on the sixteenth day of a febrile affection by no means intense; and during which there were very few symptoms of cerebral affection. On the last day of the child's life only, and that without any apparent cause, the intellectual faculties appeared completely abolished. On dissection, M. Scoutteten found the brain extremely developed, and very dense in its structure, for a child of that age, but without any other morbid appearance whatever. There was very little fluid in the ventricles.

We shall now proceed to give a condensed account of the cases which Dr. Laennec has reported in the present paper, and which may serve as materials for future inferences.

*Case* 1. Adelaide Noyau, aged 32 years, contracted a catarrh in the beginning of November, which was unattended by pain or expectoration for several days. At length some fever came on, and she was brought to Necker Hospital on the 25th November, with all the usual symptoms of bronchitis, for which she was treated with diluents and emulsions. She seemed to be getting better; but, on the 1st of December, and without any visible cause, she suddenly lost sense—the pupils became dilated—eyes fixed—left arm paralytic, right arm slightly contracted—pulse slow—respiration unequal. Twelve leeches were applied to the temples, and a blister to the nucha, with sinapisms to the lower extremities. Twelve grains of tart. antim. in the 24 hours. In the night vomiting came on. 2d December.

Strong contraction in both arms—some return of sense. 3d Dec. Answered questions correctly, but in monosyllables. 4th. All the symptoms of cerebral affection gone; but there remained an air of melancholy and inquietude about her countenance. Eighteen grains of tartar-emetic provoked no sickness. This dose (in the 24 hours) was continued for some days. On the 9th December, we find a diarrhœa and colicky pains. The dose of antimony was reduced to six grains per diem. On the 10th, the patient appeared to be going on well; but all at once fell back convulsed—breathed stertorously—and, in a few minutes, expired.

*Dissection.* The pia mater was injected, and its tissue somewhat infiltrated. The convolutions of the brain, in both hemispheres, were so remarkably flattened as to lead to the expectation that a large quantity of fluid was effused into the ventricles. The cerebral substance was singularly firm, and even elastic. The lateral ventricles did not contain quite a drachm and a half each of serum. There was about the same quantity at the base of the cranium. The chest presented no lesion worthy of notice. The mucous membrane of the stomach was covered with a thick and viscid mucus, of a rose colour. When this was scraped off, there were red and punctured stripes observable in the membrane, and some sanguineous exudation under the mucous tissue. The mucous membrane of the intestines was healthy.

*Observations.* The foregoing case presents the two principal phenomena of hypertrophia in any organ—augmentation of volume, as evinced by the flattening of the convolutions, and augmentation of density, as proved by the elasticity of the parts when cut into. It is curious that, after such quantities of tartar-emetic had been given, there should have been found such a healthy state of the mucous membrane of the intestinal canal.

*Case* 2. COLICA PICTONUM—EPILEPTIC ATTACK DURING CONVALESCENCE—DEATH IN 24 HOURS—FLATTENED CONVOLUTIONS, WITHOUT EFFUSION.

JEAN BAPTISTE, aged 44 years, white-lead manufacturer, was admitted into LA CHARITÉ on the 24th of April, with the usual symptoms of colica pictonum, and for which the celebrated treatment of LA CHARITÉ (so often noticed in this Journal) was prescribed. By the 18th May, he was cured of the colica pictonum. On the 19th he fell suddenly in a fit of epilepsy, which continued, with more or less violence, for 24 hours, when he expired. Twelve leeches had been applied to the temples; but no blood was drawn from the arm.

*Dissection.* The meninges of the brain were very pale—the surface of the convolutions remarkably flattened. On slicing the cerebral substance, no bloody points were seen, and the brain was particularly firm. There was a small portion of softened substance at the inferior part of the left hemisphere. There was scarcely any fluid in the ventricles, or at the base of the brain. There was no morbid appearance in any of the other organs of the body.

*Case* 3. This patient had also been subjected to the insalubrious influence of saturnine exhalations. He was 43 years of age—had served as a marine in early life—and latterly in a white-lead manufactory, where he became very much constipated—and, having suddenly lost power in his limbs, he was taken to the Hospital BEAUJON, where he recovered. He returned to his avocations, and again the constipation of bowels became troublesome. On the 1st January he fell down insensible, and remained so for three hours. On the 4th of the same month, a similar attack occurred, and he was carried to LA CHARITÉ, in a state of stupor, with loss of power in his limbs. The treatment for colica pictonum was put in force, and he seemed to get better; but, on the 10th of the same month, he experienced a violent epileptic seizure, succeeded by such a delirium ferox, that the strait-waistcoat became necessary. He recovered from this; but, on the 20th January, a new epileptic attack took place, followed by several other seizures of a similar kind, with intervals of profound coma. The left arm was observed to be paralytic, while the right was spasmodically contracted. On the 21st he died comatose.

*Dissection.* The meninges were slightly injected—and the convolutions of the brain were so flattened, that they appeared as one smooth surface. The ventricles did not contain a single drop of fluid. The cerebral substance was of its natural colour and consistence, and its vessels were somewhat injected. There was no other morbid appearance in the head. The heart was intimately adherent to the pericardium by membranes of old formation. (He had some severe attacks of acute rheumatism in early life.) There was no other lesion of any consequence, or that could be at all connected with the death of the patient.

*Case* 4. Subject exposed for Six Months to the Emanations of Lead—One Attack of Colica Pictonum—Attack of Epilepsy—Death—Flattening of the Convolutions—No serous Effusion.

A young woman, 22 years of age, was brought into La Charité in the latter days of February, 1828, for the treatment of colica pictonum, which was only of five days' standing. She was put on the usual plan of cure. On the 14th of March she was discharged completely well. On the 30th of the same month, she was brought by the police officers to the hospital, having been found insensible in her room, with a large ecchymosis over the left eye and temple. Her limbs were rigid and occasionally convulsed—pulse small—face pale—breathing scarcely perceptible—in short, she appeared to be at the point of death. They learnt from her friends that she had appeared quite well up to the evening before the above day, when she fell down in an attack of epilepsy, which lasted half an hour. On recovering, she asserted that she never had had any attack of the kind before. She seemed to recover a little in the hospital, during a few hours; but, on the 2d of April, she expired in an epileptic seizure.

*Dissection.* All the organs, excepting the brain, were perfectly sound. The convolutions of the hemispheres were remarkably flattened—and the substance of the brain very dense. The organ appeared to be too large for the containing cranium, and could not be got in again. The meninges were pale, healthy, and could be easily stripped off the cerebral substance. Not a drop of fluid was found in the ventricles.

The fifth case we shall not detail, as there were symptoms of hydrocephalus in a girl of 13 years of age, and an epileptic attack preceded death. On dissection, the convolutions were, as in the other cases, completely flattened; but there was a considerable quantity of water (℥iij.) in the ventricles. The cerebral substance was very firm, as was the texture of the nerves within the cranium.

The author comes to the following conclusions from the facts that have presented themselves to his observation, and from the history of cases recorded by others.

"1mo. The brain, like every other organ in the body, is susceptible of a notable augmentation of volume and density—in other words, of a veritable hypertrophy.

"2ndo. The anatomical characters of this hypertrophy consist in a very great firmness of the cerebral substance, and a flattening of the convolutions, coinciding with a vacuity or nearly a vacuity of the ventricles—sometimes a contraction or diminution of these cavities.

"3tio. This pathological condition coincides constantly with epileptic or epileptiform affections.

"4to. This hypertrophy develops itself with much more rapidity than the hypertrophy of any other organ—a circumstance which renders it similar, in many repects, to inflammatory turgescences.

5to. The causes which produce colica pictonum (emanations from lead) appear to have a strong influence in the production of hypertrops cerebri."—Rev. Medicale.

*Remarks.* There can be no reason for denying that the brain, like the heart, liver, spleen, or other parts, may be susceptible of a *morbid* augmentation (if the word *morbid* is allowable) of its *natural* structure. But if this takes place in the brain, it must be productive of much worse consequences than hypertrophy of any other organ, on account of the osseous parietes which surround the brain. Any increase of size in this delicate organization must induce the same effects, or nearly so, as pressure from increased quantity of blood circulating in the vessels at least—if not from blood extravasated out of the vessels. When we consider what anomalous symptoms present themselves to us in the living body, and how unsatisfactory are the appearances on dissection of the brain, it is not unreasonable to suppose that this pressure does sometimes exist in the head and spinal canal; but whether arising from hypertrophy of the cerebral and spinal mass, or from turgescence of their own vessels, it may be very difficult to determine.

Baron Dupuytren has made an observation which is highly worthy of notice on this occasion. He has had numerous opportunities of seeing the effects of Valsalva's method of treating aneurisms, namely, by starvation and repeated bleedings. He asserts that, "under this mode of treatment, the aneurismal tumours of the chest, abdomen, and extremities, have frequently augmented in volume, instead of diminishing—and have ended in rupture of their parietes." He explains this phenomenon by supposing that the coats of the vessels are weakened by this depletion in a greater proportion than the heart—hence the disruption of the arterial parietes by inordinate depletion. These remarks are illustrated by a case which lately occurred in the HÔTEL DIEU. The patient laboured under aneurism of the external iliac artery, situated so high up that it was found impracticable to apply a ligature *above* the tumour, and Dupuytren condemns the attempt, revived by Mr. Wardrop, of tying the vessel *ultra aneurysma*. The patient having been seized with some symptoms indicative of thoracic inflammation, repeated venesections were employed, together with the other items of antiphlogistic treatment. The aneurismal tumour, instead of diminishing under this plan, rapidly augmented, and quickly burst, with destruction of life.

We have strong reasons for believing that, in many cases of obstinate headaches, and various other anomalous cerebral affections, where depletion exasperates rather than relieves the complaints, there is a degree of pressure kept up on the brain, more by weakness of the vessels than by the vague and irrational pathological condition called "*determination of blood.*" We are led to this conclusion from the general appearance and constitution of the individual; and from the fact that bark and arsenic often remove these head-aches, vertigoes, dimness of vision, loss of memory, &c. &c. when the lancet, leeches, and purgation fail. We would strongly advise those of our brethren, who go beyond the usual routine of bleeding, purging, and blistering —and who have the curiosity to examine bodies after death, to take notice of this flattening of the convolutions *unaccompanied* by hydrocephalic distention of the ventricles. We think it must be allowed that the existence of this flattening can only be accounted for by *pressure;* and if there be no accumulation in the ventricles, we must attribute this pressure, either to distention of the vessels, or hypertrophy of the brain.

### HOPITAL DE LA CHARITE*.

**I. PARTIAL DISLOCATION OF THE KNEE.**

A cooper, 64 years of age, whilst carrying a cask filled with shavings, was thrown to the ground by a horse, and struck by the cask on the outside of the knee. He was instantly taken to the hospital, when a partial dislocation inwards of the knee was discovered. The thigh and the leg formed an angle, the apex of which was directed towards the opposite limb—the foot was separated to the distance of three or four inches from its fellow—the head of the tibia projected for about half an inch on the inside of the joint, and the external condyle of the femur to a similar extent on the outside—a good deal of fluid was effused about the head of the fibula, which was felt to be carried inwards with the tibia—and finally, the flexion and extension of the joint were excessively difficult and painful.

From the foregoing symptoms it was clear that the head of the tibia was partially dislocated inwards, and the surgical attendants proceeded to employ extension from the foot and counter extension from the hip, together with local pressure by the hands. These means not succeeding, the surgeon placed his knee against the external condyle of the femur, and pulled with all his force the head of the tibia outwards towards him, when reduction was promptly effected without any audible snap. Next morning, however, the leg being somewhat displaced again, the limb was put up in the apparatus for fractures of the thigh, and not removed for nearly three weeks. Even after that lapse of time, the leg turned out, and led to the suspicion that the internal lateral ligament was ruptured. The fracture apparatus was reapplied, the limb having previously been bandaged, and pressure was made on the inside of the knee in order to counteract the disposition to displacement. Thirty nine days after the accident, the rollers, &c. were removed, and, Jan. 20th, the date of the report, the joint had its natural form, though the patient was still obliged to use sticks to support himself in walking.

We detail this case because Sir Astley Cooper, in his notice of dislocation of the head of the tibia inwards, observes, that reduction may be readily effected by direct extension, with but little subsequent diminution of power in the joint. "The first case of the injury, which I recollect seeing," says Sir Astley, "was brought into St. Thomas's Hospital, during my apprenticeship there. I remember being struck with three circumstances respecting it ; first, the great deformity of the joint—second, the little force necessary to reduce the displacement—third, the slight degree of local or constitutional suffering which followed, the recovery being complete in a few weeks." The case we have recorded, shews how guarded a surgeon should be in delivering a decided opinion on the difficulties or facilities accompanying the reduction of any luxation, so many collateral circumstances interfering with the constant presence of either.

**II. CARIES OF THE FIRST AND SECOND CERVICAL VERTEBRÆ — DISLOCATION OF THE ATLAS—DEATH.**

Whatever be the faults of our English hospital reporters, we must say their cases are in one respect better than the French, they are not so interminably long. The following occupies just *ten* pages of the Journal Hebdomadaire, and our readers may conceive that with such *matériel* to work upon, our analytical labours are anything but a sinecure. The Interne, M. Sabatier, who reports the case, observes, with justice, that affections of the cervical vertebræ are not yet so well understood as those of the dorsal and lumbar divisions of the spine. Their comparative rarity will account for our want of very accurate information res-

---

* Journ. Hebdom. Nos. 16-17.

pecting them, and it therefore becomes a matter of importance to record the cases that occur. By this means insulated facts are amassed till a goodly store of information is obtained, from which the individual practitioner may draw wherewith to assist his diagnosis or his practice.

Francis Monçeaux, entered the army at eighteen years of age, and after being in it six years, was ordered to join some troops that were embarking. In order to elude this order he contracted a gonorrhœa, for which he was sent to the Military Hospital, and of which he was cured at the end of two months, being ordered before his dismissal, by way of precaution, two or three doses of Van Swieten's liquor. The man obtained his discharge from the army, followed his original occupation of a stone-mason, married, in ten years lost his wife, and married again. Some little time after his second marriage, the urethral discharge re-appeared, unaccompanied with any secondary symptoms, but under the use of some baths disappeared. After this, for several years in succession, Monçeaux was subject to erysipelas of the face every Spring, followed by an eruption, for which he was in the habit of purging himself with success. In 1825, an abscess formed on the index finger of the left hand, which produced considerable inflammation and swelling of the hand, fore-arm, and arm as high as the shoulder. Scarcely had this passed away, when the legs became œdematous, and the neck became the seat of pains which were aggravated on motion of the head, and soon compelled him to give up his usual employment. He applied to a physician who considered the case as one of a syphilitic nature, and salivated the patient, without producing the least relief. In Jan. 1828, an abscess formed at the upper border of the sternum, and shortly afterwards lateral curvature of the spine commenced, with inclination of the head to the left side. The abscess on the sternum was opened, and carious bone exposed, when the unhappy patient was again injuriously submitted to a second mercurial course. In August, 1828, an abscess formed in the tonsil, which, before it was opened, produced urgent symptoms, and towards the termination of the month he became affected with contraction and trembling of the jaws, difficulty of deglutition, and violent pains in the head. Leeches to the base of the cranium, frictions to the neck, sinapisms to the feet, and the vapour bath were now had recourse to but without the slightest good effect, and another physician recommended "flying blisters" to the nucha. His strength, however, was exhausted, and his family brought him to La Charité, on the 13th of last December, in a very deplorable condition. He was now fifty-four years of age.

In front of the sternum was an opening leading to carious bone—the head was immoveably declined towards the left, on which side the cervical region presented a remarkable inflexion — no tumour was perceptible externally, nor any projection of the cervical spines—the act of drinking was extremely difficult, but that of mastication was next to impossible on account of the pain which it occasioned —the patient had not been able for the last two months, to assume any other position than that of decubitus on the left side—the upper and lower extremities were freely moved—the articulation was slow—the appetite weak—the bowels obstinately costive — the emaciation extreme. For some months cough had been present, and he expectorated a quantity of thick, and apparently purulent matter. Caustic issues were applied, and on the 1st January, he complained of more pain than usual in the neck, which he attributed to his having made a trifling exertion. At nine, a. m. after speaking to one of his neighbours, he suddenly lay upon his back, his head sank forward, some mucus escaped from his mouth, and he instantly expired.

*Dissection.* We cannot afford space for the minute description of the morbid appearances furnished by M. Sabatier, and shall therefore give only the principal. The anterior arch, or body of the atlas, was carious through almost its whole extent—the right side of the bone was nearly destroyed altogether—the superior and inferior articulating surfaces on this side were gone, and the corresponding condyle of the os occipitis was denuded of cartilage, and in part destroyed. The left side of the bone, and left condyle of the occipital were free from alteration. The posterior arch of the atlas on its right side was scarcely a line in thickness, and the hole for lodging the vertebral artery was compressed by the condyle of the occipital bone to a sixth of its natural size. The body of the dentata was carious in front, more deeply still on its right side, not quite so deeply on its left. The superior articular process on the right side was in part destroyed, and the transverse process was completely separated, and hung by some ligamentous shreds. The odontoid process was extensively but superficially carious, the ligaments attached to its apex were destroyed, the transverse still in part remained. The atlas was dislocated upon the dentata, for the ligaments uniting their articulation on the *right* side being gone, the atlas had been dragged towards the left, the head had followed in the same direction, and the ligaments on this side had been put upon the stretch. The right condyle of the occipital bone finding no support had pressed on, and during the exertion of the patient had finally separated the transverse process of the atlas, in consequence of which, the odontoid process had projected upwards beyond the upper border of the atlas, and compressed in front the commencement of the medulla oblongata, so as to produce the patient's death.

The inferior surface of the dentata, the third cervical vertebra, and the intervertebral cartilage between them were more or less diseased. The transverse processes of the 4th, 5th, and 6th, cervical vertebræ, were affected, and connected with the 5th, was a small abscess communicating through the vertebral canal of the 6th, with a larger one below. From the level of the upper border of the 4th cervical vertebra, this *foyer* extended down to the junction of the 2d and 3d dorsal, where it ended in a kind of pouch, bounded in front by the thickened pleura, through which it communicated with a "spacious cavern" in the lung, half filled with pus. Into this cavern, which, as well as the pouch, was lined with "an organized mucous membrane," several bronchial ramifications opened. The right vertebral artery was greatly diminished in bulk, evidently due to the compression it had undergone—the left was equal in size to the external carotid. The spinal marrow was somewhat flattened opposite the atlas from left to right, and the odontoid process had left a very sensible depression on the medulla oblongata.

As M. Sabatier, observes, the principal features of this interesting case were first of all, general pains of a rheumatic character disappearing at the end of six months—several years after this, fixed pains in the neck, extreme difficulty in the motions of the head, constant pain in the left arm and shoulder, painful mastication, and difficult deglutition—lastly, declination of the head towards the left, violent and almost continual cephalalgia, absence of palsy of the limbs. The sudden death of the patient from displacement of the odontoid process, renders the case somewhat similar to those recorded by Mr. Bell, in his Observations on Injuries of the Spine, though in those the principal disease appears to have existed in the ligaments. Had it not been for the general cachectic state of the patient's system, a state materially aggravated, if not called into existence by the successive and ruinous mercurial courses, we see no reason why the disease should not have ended in bony anchylosis. The principal disease was betwixt the atlas and occipital bone, and the number of cases of anchylosis of these bones after dislocation, afford, as Mr. Lawrence has well observed, not only a proof that the affection admits of cure, but that the prognosis cannot be very unfavourable. We have witnessed two cases, one a very marked one, where every reason existed for supposing that disease of the atlas or dentata had gone to some extent, and yet the patients got perfectly well.

A symptom was present in this case, which we think will be found by no means an uncommon one, although it has neither been alluded to by Rust, nor his translator, Mr. Lawrence; we mean, the most violent pain in the head. The pain is by no means confined to the occiput, but darts to the side of the head, and more especially the forehead. This is sometimes the symptom which absorbs the whole of the patient's attention, and if not understood might put the practitioner on the wrong scent as to the seat of the disease. Mr. Brodie, we believe, has seen one or two cases, where the pain in the head was extremely severe.

III. Absence of the Menses—Encysted Abscess in the Uterus.

However opposed we may be to the ridiculous notion of making obstruction of the os uteri the cause of dysmenorrhœa, still no doubt can exist that the absence of the menstrual discharge does, in a few rare cases, depend on mechanical impediments to its flow. The knowledge of the fact should induce the practitioner, in particular cases, to institute a manual examination.

A young woman, twenty-one years of age, who had never experienced the menstrual flux, nor felt the symptoms generally precursory of its first appearance, died in La Charité of a lumbar abscess, connected with caries of one of the vertebræ of the loins, as well as disease of the os ilii. On examining the uterus, which was larger than usual, it was found to be accurately filled by a cyst, which presented no outlet either at the neck of the womb, or the mouths of the fallopian tubes. It adhered to the uterus, but was easily detached, and contained within it three or four ounces of yellowish grey pus. Towards the posterior part of the organ, the cyst was eaten through by two ulcerated spots, the bottom of which was the substance of the viscus. No trace remained of the mouths of the fallopian tubes, and a bristle passed into their cavity was stopped near the uterine cavity, which it could not be made to enter. The calibre of the tubes themselves was considerably diminished, and at their loose ovarial extremities they were sealed as completely as they were at their uterine. The surface of the ovaries presented the marks of several cicatriculæ, and both contained a corpus luteum. Several little hollow, ovoid bodies, existed in the fallopian tubes and within the layers of the broad ligament.

The author of the case, M. Reynaud, subjoins some rather lengthy reflections, but the naked facts are all that we shall give, and all indeed that are required, for each of our readers may form what conclusions he pleases.

---

